# PW02-024 - A case of candle syndrome treated with thalidomide

**DOI:** 10.1186/1546-0096-11-S1-A164

**Published:** 2013-11-08

**Authors:** K Yamazaki, T Miyamae, S Yokota

**Affiliations:** 1 Pediatrics, Yokohama City University, Yokohama, Japan

## Introduction

A new group of autoinflammatory diseases caused by immunoproteasome dysfunction has been recently reported. The mutation in the PSMB8 gene encoding immunoproteasome subunit β type 8 causes a number of clinical syndromes that described as chronic atypical neutrophilic dermatosis with lipodystrophy and elevated temperature (CANDLE) syndrome and Nakajo-Nishimura syndrome (NNS).

## Case Report

A Japanese girl presented with fever, annular erythematous plagues and elevation of hepatocellular enzyme at 2 months of age. She had deformed ears, a broad saddlelike nose and periorbital edema. At 16 years of age, she had lipodystrophy of the face and upper limbs, a protuberant abdomen, and severe fat deposition into the peritoneal and the pleural cavity. Painful nodular erythema, hepatosplenomegaly, muscle atrophy, mild joint contracture of ankle, and mild mental retardation were observed. She suffered from arthralgia without arthritis for her lifelong. Laboratory findings showed hypochromic anemia, and elevation of erythrocyte sedimentation rate and C-reactive protein. A brain computed tomographic image revealed basal ganglia calcification. After obtaining informed consent, the patient’s DNA was analyzed for mutations in PSMB8, and heterozygous c145C>A mutation (Q49K) was found. She was unsuccessfully treated with NSAIDs, a variety of immunosuppressants: cyclosporine, tacrolimus and mycophenolate mofetil, and biologics: infliximab and tocilizumab. Pulsed intravenous methylprednisolone and high dose of oral prednisolone (PSL) were effective. Thalidomide had an efficacy for improvement in her symptoms and reduction of PSL dosage, but the treatment had to be terminated due to thrombocytopenia.

## Discussion

We reported here the case of severe CANDLE syndrome. Although both her disease course and clinical presentations were typical, only heterozygous Q49K mutation in the PSMB8 gene was found. Recent studies showed that thalidomide combined with statin was involved in anti-myeloma action by p38 MAPK inhibition, as well as thalidomide inhibits lipopolysaccharide-induced tumor necrosis factor α production. The efficacy of thalidomide in our case indicates that thalidomide could regulate the inflammatory signaling induced by immunoproteasome dysfunction, and thalidomide might become key treatment other than PSL.

## Disclosure of interest

None declared.
